# Assessment of Amyloid Forming Tendency of Peptide Sequences from Amyloid Beta and Tau Proteins Using Force-Field, Semi-Empirical, and Density Functional Theory Calculations

**DOI:** 10.3390/ijms22063244

**Published:** 2021-03-23

**Authors:** Charuvaka Muvva, Natarajan Arul Murugan, Venkatesan Subramanian

**Affiliations:** 1 Division of Theoretical Chemistry and Biology, School of Engineering Sciences in Chemistry, Biotechnology and Health, KTH Royal Institute of Technology, S-106 91 Stockholm, Sweden; charvakmuvva@gmail.com; 2 Inorganic & Physical Chemistry Laboratory, CSIR-Central Leather Research Institute, Adyar, Chennai 600020, India; subbu@clri.res.in; 3 Academy of Scientific and Innovative Research (AcSIR), Ghaziabad 201002, India; 4 Centre for High Computing, CSIR-CLRI, Adyar, Chennai 600020, India

**Keywords:** Alzheimer’s disease, amyloid-β peptide, amyloid forming peptides, Tau protein, umbrella sampling simulations, free energy calculations, QM calculations

## Abstract

A wide variety of neurodegenerative diseases are characterized by the accumulation of protein aggregates in intraneuronal or extraneuronal brain regions. In Alzheimer’s disease (AD), the extracellular aggregates originate from amyloid-β proteins, while the intracellular aggregates are formed from microtubule-binding tau proteins. The amyloid forming peptide sequences in the amyloid-β peptides and tau proteins are responsible for aggregate formation. Experimental studies have until the date reported many of such amyloid forming peptide sequences in different proteins, however, there is still limited molecular level understanding about their tendency to form aggregates. In this study, we employed umbrella sampling simulations and subsequent electronic structure theory calculations in order to estimate the energy profiles for interconversion of the helix to β-sheet like secondary structures of sequences from amyloid-β protein (KLVFFA) and tau protein (QVEVKSEKLD and VQIVYKPVD). The study also included a poly-alanine sequence as a reference system. The calculated force-field based free energy profiles predicted a flat minimum for monomers of sequences from amyloid and tau proteins corresponding to an α-helix like secondary structure. For the parallel and anti-parallel dimer of KLVFFA, double well potentials were obtained with the minima corresponding to α-helix and β-sheet like secondary structures. A similar double well-like potential has been found for dimeric forms for the sequences from tau fibril. Complementary semi-empirical and density functional theory calculations displayed similar trends, validating the force-field based free energy profiles obtained for these systems.

## 1. Introduction

Protein aggregation is essentially a self-association process in which many identical peptides form higher-order conglomerates of low solubility that eventually precipitate [1,2,3]. Protein aggregation underlies the pathogenesis of several human neurodegenerative diseases, such as Alzheimer’s (AD) [4,5], Parkinson’s (PD) [6,7], Huntington’s diseases (HD) [8,9], prion [10,11], Amyotrophic Lateral Sclerosis (ALS) [12,13], and Frontotemporal lobar degeneration (FTLD) [14]. All of these diseases have common cellular and molecular mechanisms including protein aggregation and self-amplifying inflammatory processes. In all these cases, certain protein fragments misfold and assemble to form amorphous structures that can rearrange into highly organized fibrillar aggregates, so-called amyloid plaques [15]. The deposition of the aggregates in different regions of the brain leads to amyloidosis of the central nervous system [16].

The extracellular amyloid-β (Aβ) plaques and intracellular neurofibrillary tangles (NFTs) are two types of assembly of molecules that contribute pathologically to the degradation of the neurons in the brain and the subsequent symptoms of Alzheimer’s disease (AD) [17,18,19] such as cognitive impairment and memory loss. Amyloid plaques are made of amyloid-β peptides while tau peptide sequences are the components of neurofibrillary tangles. The Aβ monomers self-aggregate into different forms of oligomers, which can then form regular fibrils. These fibrils are large, insoluble, and further assemble into amyloid plaques. Over the last decade, advances have been made in understanding the structure of the Aβ peptide in both monomeric and aggregate forms. Talafous et al. indicated that the Aβ peptide (1-28) folds into an α-helical structure in normal conditions, while it adopts a β-sheet like structure in membrane-like media that may also occur during the early stage of amyloid formation [20]. Coles et al. reported that the Aβ peptide (1-40) in solution is unstructured between residues 1 and 14 and that the C-terminus residues have an α-helical conformation between residues 15 and 36 with a kink or hinge at 25-27 in aqueous sodium dodecyl sulfate (SDS) micelles [21]. Källberg et al. suggested that when the Aβ peptide residues 14-23 are removed or changed to a non-discordant sequence, fibrils are no longer formed [22]. This suggests that there exist certain amyloid forming sequences, which are responsible for protein misfolding and subsequent aggregation. The MD simulation studies on Aβ peptides from (1-40) and (1-42) proposed that the C-terminal residues of Aβ1-42 are more structured and 31-34 and 38-41 form a β-hairpin that leads to a reduction in the flexibility of C-terminal residues [23,24,25]. The replica-exchange molecular dynamics (REMD) simulations conducted by Davis et al. reported that in presence of lipid bilayer, the abeta monomer did not convert into beta-sheet suggesting that beta-sheet formation is due to protein-protein interaction [26].

Over the years, many of such amyloid forming sequences have been identified and reported in different proteins. For example, GAVVTGVTAVA (residues from 68-78) has been reported to be the sequence responsible for the aggregation of α-synuclein [27,28,29]. Similarly, AEVVFT and TAVVTN were reported to cause amyloidosis in human transthyretin [30], while ANFLVH (13-18) and NFGAILS (22-28) induce aggregation in the islet amyloid polypeptide [31,32]. In the case of the amyloid-β protein, the KLVFFA sequence has been recognized to be amyloid forming along with others such as GAIIGL (29-34) and GGVVIA [33]. In the case of the tau microtubule-binding protein, the QVEVKSEKLD and VQIVYKPVD were reported to be amyloid forming sequences [34]. Interestingly, even for proteins like lysozyme [35]., hemoglobin [36], albumins [37], and prion [38] proteins there exist certain amyloid forming sequences which serve as a nucleation center for the aggregation process contributing to the malfunctioning of proteins. Even though, if many of such amyloid forming sequences are being reported there is no clear understanding why such sequences tend to form aggregates. Such knowledge can evidently be of great help to develop amyloid aggregation inhibitors which can slow down or prevent the aggregation process at the molecular level. It is the purpose of this article to contribute to such knowledge and to better understand why the amyloid forming sequences such as KLVFFA from the amyloid-β precursor protein and QVEVKSEKLD and VQIVYKPVD from the tau protein form aggregates.

The main objective of the current manuscript is to understand why certain amyloid forming sequences from amyloid and tau proteins prefer to form β-sheet rich aggregates. We estimate the free energy profile for the α-helix to β-sheet interconversion in the case of monomer and dimeric units of these peptide sequences using umbrella sampling molecular dynamics simulations. Since the standard molecular dynamics simulations cannot capture conformational interconversion, which is generally a long time scale process, we have adopted this procedure. It has been earlier established that the average psi (<ψ>) of the peptide sequence can be used as a reaction coordinate to drive the peptide sequence to adopt various secondary structures such as α-helix, random coil, and β-sheet [39,40,41]. Following this notion, we have used the <ψ> as a reaction coordinate to obtain the free energy profile for the α-helix to β-sheet interconversion. Further, to validate the results, we also carried out density functional theory (at M06-2X/6-31+G* level of theory) and semi-empirical PM7 calculations [42] for various independent configurations along the free energy profile. In particular, due to the computational cost associated with density functional theory, it has been carried out only for the dimeric form of QVEVKSEKLD tau peptide sequence. As we will show later, the semi-empirical level of theory-based energy profile is in good agreement with this DFT based results and so for the remaining systems PM7 level of theory has been employed. As a control, the same set of umbrella sampling simulations were carried out for penta-alanine which is not amyloid forming in normal physiological conditions. In particular, alanine is referred to as helix former and alanine rich peptides are known to form alpha-helices [43] Similar to other peptides, here too, we studied monomer and dimeric forms of penta-alanine. As the system size is relatively smaller in this case, we have estimated an energy profile along <ψ> by employing both PM7 semi-empirical theory and density functional theory calculations with MO6-2X functional [42] In general, the density functional theory is known to describe dispersion interactions very poorly and so either dispersion corrected density functionals [44] or the Minnesota functionals [45,46] should be used in such systems where dispersion interactions are dominant. As in the dimers of peptides in alpha-helix secondary structures, these are dominant stabilizing interactions, so we have rightly used the DFT theory with Minnesota functional [45,46].

Overall, the energetics of interconversion were estimated using three different approaches: (i) Wham analysis of MD trajectories with umbrella sampling, (Equation (1)) (ii) g_energy which is a module for doing analysis of intramolecular and intermolecular energies within a peptide or between the peptides. In Equation (2), we present how the total energies are presented for monomeric and dimeric forms of peptides. In particular, the g_energy module allows to compute the van der Waals and electrostatic interactions within a peptide and between peptides. The total energies as a function of <ψ> were computed as the sum of van der Waals and electrostatic interactions and (iii) total energies were obtained using PM7 wave functions and DFT theory. The monomer and dimer coordinates were obtained from umbrella sampling simulations and the calculations were carried out for 10 configurations corresponding to each <ψ> (over all 190 such calculations are carried for each system) and angles were chosen at an interval of 20°. The total energies were averaged for configurations corresponding to different <ψ> and so the results on total energies are presented as a function of ψ.

(1) $$A\left(x\right)\;=\;-k_{B}T\;lnP^{\prime }\left(<\psi >\right)\;-\;U^{\prime }\left(<\psi >\right)\;+\;F$$

(2) $$E_{Total}\left(<\psi >\right)\;=\;E_{vdw}\left(<\psi >\right)\;+\;E_{elec}\left(<\psi >\right)$$

<ψ> = average psi,

$U^{\prime }$ = sample with umbrella potential

*P*′ = compute biased probability.

*F* = depends on $U^{\prime }$ (<ψ>)

*E_vdw_* = van Der Waals interactions

*E_elec_* = electrostatics interactions

## 2. Results and Discussion

**Figure 1:**
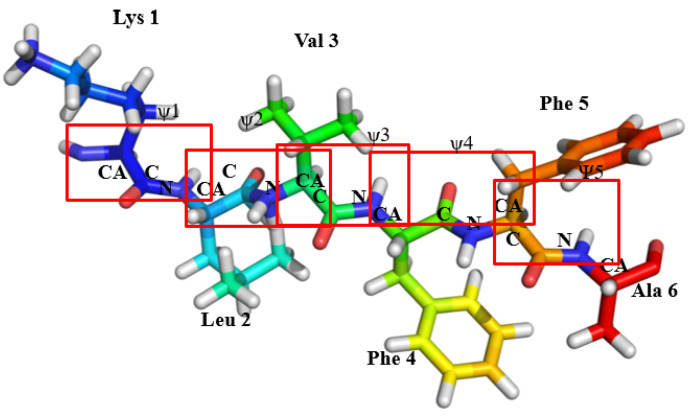
The schematic represents of monomeric form of KLVFFA, with 5 ψ angles in total with one corresponding to each peptide bond. The red box indicates the location of different peptide bonds in the KLVFFA sequence.

**Figure 2:**
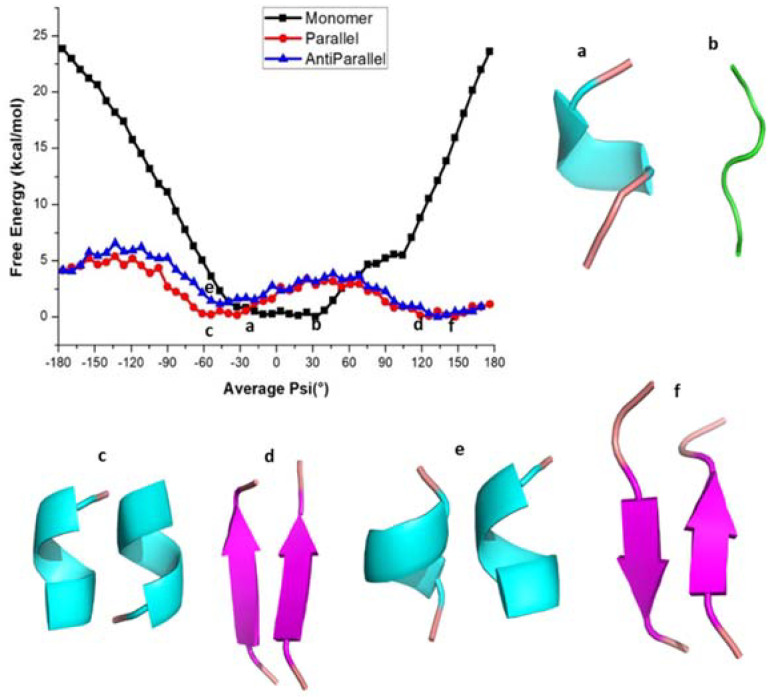
Free energy profile of various conformations of KLVFFA. (**a**,**b**) helical and coil forms of monomer, (**c**,**d**) helical and β-sheet forms of Dimer(parallel form), (**e**,**f**) helical and β-sheet forms of Dimer (anti-parallel form).

The free energy profiles along the reaction coordinate, <ψ>, for amyloid forming sequence KLVFFA (Figure 1) in different forms (monomer, parallel, and anti-parallel) calculated by the umbrella sampling method, are shown in Figure 2. In particular, the <ψ> has been computed as an average over the backbone ψ angles (−180° to 180°) of all peptides in the sequence and is considered as the reaction coordinate to study interconversion between different conformations with secondary structures such as α-helix, coil-like, and β-sheet. It has already been demonstrated in previous works that this reaction coordinate is suitable to achieve the interconversion between α-helix to β-sheet in dimers of peptides [40,41] and in certain amyloid forming prion proteins [39] The free energy profile scan reveals that the monomer (KLVFFA) has a flat minimum, whereas parallel (KLVFFA) and antiparallel (KLVFFA) dimer forms exhibit two minima on the free energy surface separated by a small energy barrier (Figure 2). To observe the structural changes along the free energy surface, we analyzed secondary structures using DSSP on trajectories from umbrella sampling simulations. The results are given in Table S1. The free energy profile of the monomer (KLVFFA) reveals that a rather flat energy minimum is found between −30° to 30° <ψ> angle and that at this minimum the peptide exists in helix and coil conformations (Table S1 and Figure 2a,b), whereas for other ψ angles secondary structures, such as coil, and bend, are dominant (Table S1). This observation suggests that when KLVFFA exists as a monomer the α-helix and coil-like structures are the preferable secondary structures (Figure S1). Even though one can appreciate the stability of an α-helix like secondary structure; it is a surprise that the coil-like structure as well corresponds to a minimum in free energy surface. In order to understand this, we have visually analyzed the structures corresponding to these ranges of average ψ angle and the secondary structure is found in α-helix for the range from −30° to 0° We find that it adopts a coil-like structure for the angle ranging from 10° to 30° It is interesting to notice that the coil-like structures have also comparable free energies like the α-helix for this monomer and so we carefully analyzed the stabilizing intramolecular contacts to explain this. There were two hydrogen bonds observed in the case of the monomer with a helical structure. We also find stabilizing interactions in the case of coil-like structures (hydrogen bonding interactions between residues Phe4 and Leu2 with Phe5 and *π-π* interaction between the residues Phe4 and Phe5). The structures of the monomer for different <ψ> angles and stabilizing interactions are included in the supporting information (as Figure S1).

In the case of dimers, two minima have been observed with the first minimum appearing at the average ψ angle −50° in both parallel and antiparallel conformations (Figure 2). The potential energy profile around this minimum is rather flat and the <ψ> angles span the range −70° to 20° in the case of the parallel dimeric form, while they span −80° to 20° in the case of the antiparallel form. The DSSP (secondary structure) analysis shows that the secondary structure adopts the α-helix form in this angle range (Table S1). The second minimum occurs between 120° to 150° (Figure 2) and the corresponding secondary structure resembles a β-sheet in both the parallel and antiparallel forms of the dimer (Table S1 and Figure 2d–f). Furthermore, as can be seen from Table S1, for both cases, the β-sheet structures appear for the <ψ> angle ranges from 40° to 180° and 50° to 180°, respectively (Table S1). The α-helix and β-sheet of parallel and antiparallel conformations are separated by energy barriers of 3.0 kcal/mol and 2.7 kcal/mol, respectively, and the intermediate structures have random coil-like structure. The maximum standard error for the free energy values at different <ψ> angles was < 0.05 kcal/mol suggesting that the reported free energy values are reliable. Stabilities of the α-helix and β-sheet observed above are also reflected in the RMSD (Figure S2). The probability distributions of instantaneous dihedral angles (there are five such ψ angles there) along the reaction coordinate for the KLVFFA monomer are shown in Figure S3 and we can observe overlap of values for different reaction coordinates which is required for obtaining a smooth free energy profile.

To get a better understanding of the energetics of different secondary structures, we carried out hydrogen bond analysis using the g_hbond command of GROMACS. The number of intra and intermolecular hydrogen bonding interactions along the <ψ> angles for the KLVFFA sequence in its different forms is shown in Figure S4. In the monomer case, the maximum number of intramolecular hydrogen bonding interactions are observed for ψ angles ranging from −40° to 20°, which can be associated with the helical formation (Table S1 and Figure S4). In the parallel form, a maximum number of intermolecular-hydrogen bonds is observed between the ψ angles −180° and −120° which can be ascribed to the presence of a β-sheet (Table S1). This reveals that the β-sheet structures of KLVFFA dimers are stabilized by intermolecular-hydrogen bonds while the helical structures are stabilized by intramolecular-hydrogen bonds. It is clear from Figure S1 that for the monomer the helical form is more stable than the coil and bend forms due to the possibility that this helix can make intramolecular hydrogen bonds. However, in the case of parallel and anti-parallel dimeric structures, the β-sheet structure is found to be more stable due to the possibility to make many intermolecular hydrogen bonds. This observation clearly explains that when the KLVFFA sequence is part of unprocessed wild-type amyloid-β protein, it exists in α-helix form, but that in the vicinity of other KLVFFA fragments (formed due to subsequent action of β-secretase and gamma-secretase enzymes) it tends to form aggregates which can grow and make insoluble amyloid plaques contributing to the disease pathology in Alzheimer’s disease.

**Figure 3:**
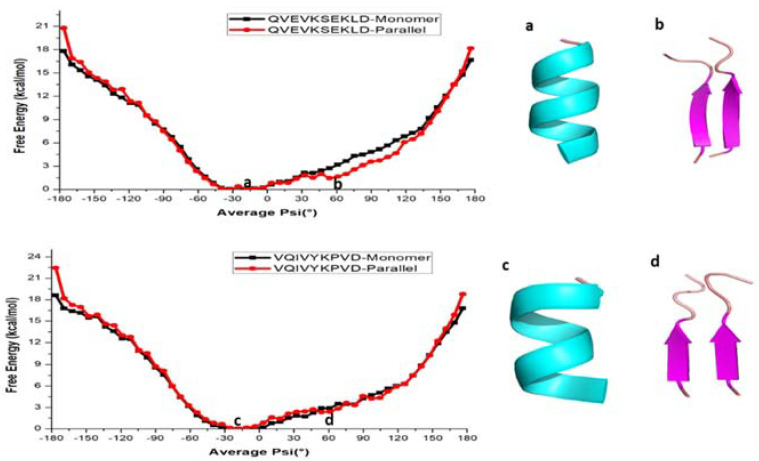
Free energy profile of various conformations of QVEVKSEKLD and VQIVYKPVD respectively. (**a**,**b**) helical and β-sheet of QVEVKSEKLD, (**c**,**d**) helical and β-sheet of VQIVYKPVD.

It is also of interest to see whether the above finding is common for all amyloid forming sequences from other proteins such as the tau protein. We also calculated the free energy profile along the reaction coordinate, <ψ> for the monomer and dimer forms of QVEVKSEKLD and VQIVYKPVD peptide sequences from tau protein and the results are represented in Figure 3. As shown, the free energy profile contains one minimum at −30° in both monomers (Figure 3a,c) of tau (QVEVKSEKLD and VQIVYKPVD), whereas the parallel forms of tau, two minima at −30° and 60° (Figure 3b,d) appear. It is notable, compared to the case of KLVFFA that the second minimum for the dimers is not very deep suggesting that even in the case of the dimer, α-helix like structure corresponds to the global minimum. The monomer of QVEVKSEKLD (−80° to 20° helix) and VQIVYKPVD (−70° to 20° helix) systems reflects the formation of a helix for <ψ> angles ranging between −80° and 20°, while for other ψ angles, secondary structures are dominated by coil and bend like structures (Tables S2 and S3). For the dimers of QVEVKSEKLD and VQIVYKPVD, helical conformations are observed for angles ranging from −90° to 20° and −70° to 20°, respectively (Tables S2 and S3). Furthermore, one can observe β-sheet like structures in the angle range from −130° to −180° and from 100° to 180° for QVEVKSEKLD (Table S2). For the dimeric form of VQIVYKPVD, β-sheet like structures are observed for the angles from −120° to −180° and from 130° to 170° (Table S3). In contrast to the case of KLVFFA, the free energy barrier for α-helix to β-sheet interconversion is not significant (Figure 3).

We further investigated the role of hydrogen bonding interactions in the total energetics of both monomer and dimeric forms (Figure S5). The analysis reveals that the monomer form of QVEVKSEKLD (corresponding to the <ψ> ranging from −80° to 20°) and VQIVYKPVD (corresponding to the <ψ> ranging from −70° to 20°) shows a relatively large number of hydrogen bonds, reflecting the formation of helical structures (Tables S2 and S3). It is relevant to repeat here that the α-helix structures are stabilized by intramolecular hydrogen bonds. In the parallel forms, the intermolecular hydrogen bonds are observed for the structures having ψ angles between −130° to −180° and 100° to 180° for the QVEVKSEKLD dimer which can be associated to the formation of β-sheets (Table S2). In the case of the VQIVYKPVD dimer, the β-sheets are observed for the dihedral angle ranging from −120° to −180° and 130° to 170° (Table S3). Similar to the case of KLVFFA, the maximum number of intermolecular hydrogen bonds is observed for the β-sheet structure, while intramolecular-hydrogen bonds are more present in the helical forms for both the QVEVKSEKLD and VQIVYKPVD dimers.

Now we will discuss the energetics of interconversion using total energies and intermolecular energies as computed using g_energy. The above discussion is based on the free energies, which are computed from the probability distribution of certain degrees of freedom (<ψ> in our case). Additionally, one can compute the total energies and intermolecular energies for the configuration of monomers and dimers respectively which are collected from the MD trajectories carried out at different <ψ> values. Further, the total and intermolecular energies can be computed as a function of <ψ> and can be analyzed. The total energies for monomers and intermolecular energies for dimers as a function of average ψ were computed and the results are presented in Figure S6. The total energy, that is the sum of van der Waals and electrostatic contributions, as a function of the average ψ angle for KLVFFA monomer shows a single well potential (Figure S6a). This is consistent with the free energy profile as obtained from a WHAM analysis (Figure 2).

In Figure S6b, the intermolecular energy calculated for the parallel and antiparallel is presented for the KLVFFA sequence. Further, the minimum in energy is corresponding to the <ψ> angle ranging from 90° to 150°, which attributes to β-sheet like secondary structures. In the same way as we did for the KLVFFA sequence, the total and inter-molecular energy of the monomer and dimer of the tau sequences were computed (Figure S7) and the results displayed a similar pattern as that of KLVFFA. It is clear from Figure S7a that the total energy (magnitude) is higher and appears at around ψ angles from −80° to 20° for the QVEVKSEKLD monomer and −70° to 20° for the VQIVYKPVD monomer which is again consistent with free energy profile obtained from the WHAM analysis. Figure S7b shows the intermolecular energy of both dimers of the tau protein which displays a minimum around the ψ angle 90 and this has to be attributed to the maximum number of intermolecular hydrogen bonds formed for β-sheet like structures. Interestingly, the intermolecular energy plot for dimers of sequences from tau protein displays a deep minimum for the <ψ> angles in the range 50° to 150° where the WHAM based free energy profile only shows a shallow minimum. This analysis of the energetics of the monomer and dimer of peptide sequences from the tau protein suggests that for the monomer an α-helix structure is the preferred one while in the vicinity of a neighboring peptide sequence, a β-sheet like structure is preferred. This may be a general feature for all amyloid forming sequences.

**Figure 4:**
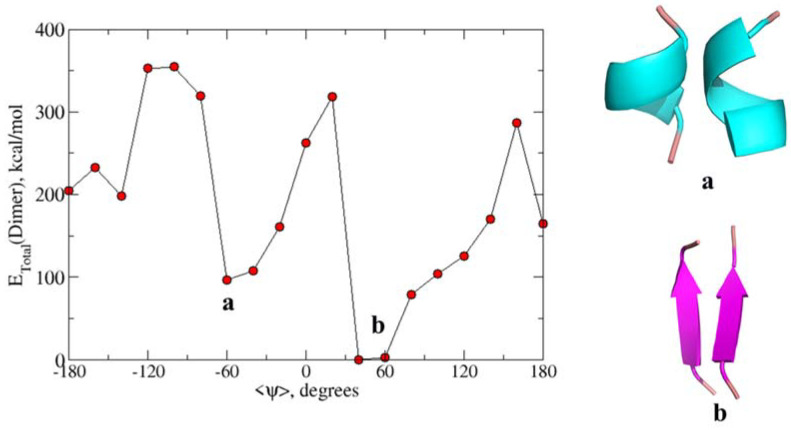
Calculated total energy of conformations at different ψ angles using the M06-2X/6-31+G* level of theory for QVEVKSEKLD. (**a**) The secondary structure corresponds to the alpha helix dimer and (**b**) Beta-sheet dimer.

To additionally validate the free energy profiles of peptide sequences from the tau proteins as obtained from the force-field methods, we employed density functional theory. In particular, MO6-2X level of theory and 6-31+G* basis sets have been used to estimate the total energy of dimers of the QVEVKSEKLD peptide. There were 10 calculations carried out for dimers having <ψ> angles in the range −180° to 180° (with the increment of 10 degrees). The total energies averaged over dihedral angles are plotted in Figure 4. As can be seen, the figure displays two minima respectively at dihedral angles −60° and +50° which correspond to alpha-helix dimer and beta-sheet dimer. Further, the energy for the latter type of dimer is lower than that for the former suggesting that for this tau sequence beta-sheet like secondary structure is preferable in the dimeric form than in the alpha-helix form. The energy difference between the two forms is closer to 100 kcal/mol and such an overestimate has to be attributed to the neglect of the solvation effect in these calculations. As we will show that the semiempirical PM7 based calculations for this dimer also showed the same behavior in the energy profile, we have adopted this approach to study the energy profiles for all the peptide sequences that are studied in this article.

**Figure 5:**
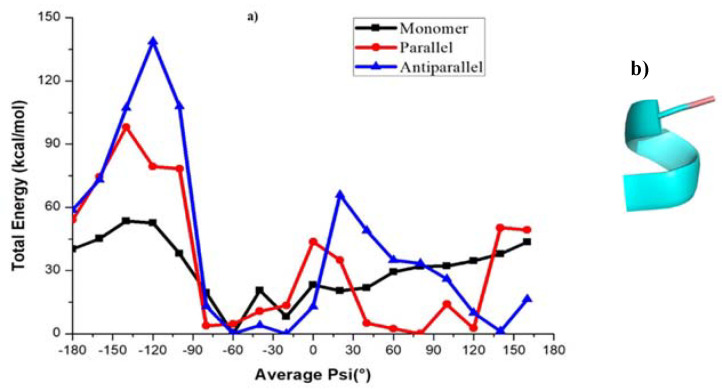
(**a**) Calculated total energy of conformations at different ψ angles using the PM7 level of theory for KLVFFA. (**b**) The helical conformation at first minimum (−60°) of KLVFFA.

Using the semiempirical PM7 approach, the energies were calculated for 20 configurations corresponding to different <ψ> angles and the values were averaged to get the total energies as a function of <ψ> angles. Figure 5 depicts the energy profile as a function of the <ψ> angle for monomer and dimers of KLVFFA. It is worth recalling that this energy profile is based on semi-empirical PM7 wave functions while the previous results are based on the force-field methods.

**Figure 6:**
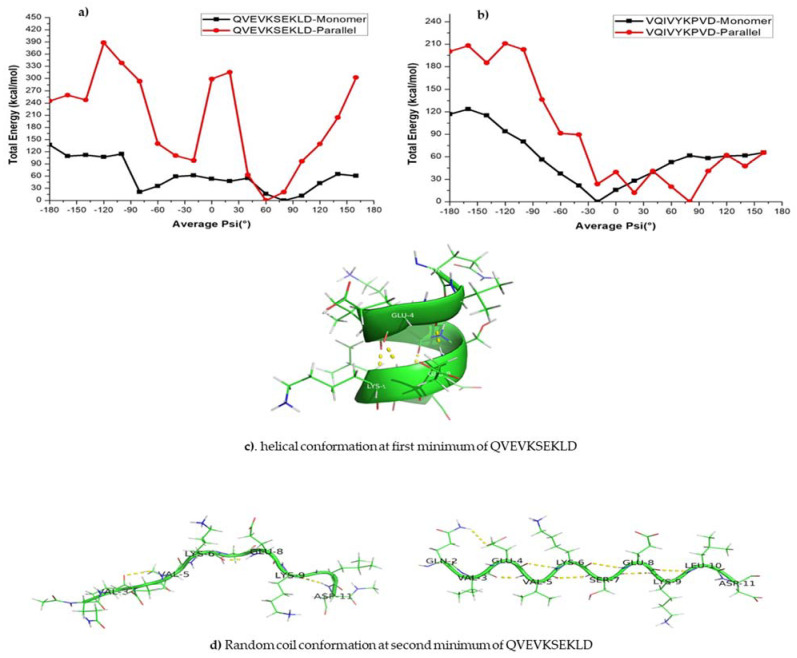
(**a**,**b**) Calculated total energy of conformations at different ψ angles using the PM7 level of theory. (**c**,**d**) The secondary structure that corresponds to the first minima and second minima respectively.

The energy profile of the KLVFFA monomer shows a minimum at −60°. The structure that corresponds to the first minima is mostly characterized by a turn and a closer look suggests that the secondary structure is helical (Figure 5a). The dimers exhibit a double well potential corresponding to the secondary structures, namely α-helix and β-sheets. The energy profile for the KLVFFA sequence as we get from the PM7 level of theory is fully consistent with the free energy profile obtained from the WHAM analysis and supports the reliability of the force-field method. Similarly, we have plotted the energy profiles for monomer and dimers of tau peptide sequences. Comparable to the KLVFFA sequence, the monomer of VQIVYKPVD displays a single minimum in the energy profile (Figure 6b). The minimum corresponds to ψ −20° and the secondary structure can be easily attributed to α-helix from visual analysis. The dimeric form of VQIVYKPVD displayed a rather rugged energy profile along the <ψ> angle and the single minimum appeared at ψ angle 70° that can be easily associated with β-sheet like secondary structure. Interestingly, the QVEVKSEKLD monomer displayed a very different energy profile compared to other peptide sequences studied here. It rather exhibited a double well potential and the two minima appeared at ψ angles −80° and 80°. The secondary structure at the first minimum of QVEVKSEKLD corresponds to helix, whereas the second minimum of QVEVKSEKLD corresponds to a linear conformation (Figure 6d). The reason for the stabilization of this conformation in the monomeric state is due to the dominant electrostatic interaction between adjacent negatively (Glu4, Glu8) and positively (Lys6) charged residues. This explains that for monomeric forms of all peptide sequences, it is not necessary that the helical structure is the most stable one and the preference of a specific secondary structure depends on the nature of the amino acid composition. When it comes to the dimeric form of this sequence, there are not any more surprises as the energy profile displayed double well corresponding to α-helix and β-sheet like structures. The β-sheet like secondary structure appears to be the one with the least energy and so corresponds to the global minimum. In contrast to the case of KLVFFA the energy profile displayed very different features when compared to the free energy profile obtained from the WHAM analysis. However, the agreement between the energy profile obtained from PM7 semi-empirical calculations and the total energy profile and intermolecular energy profile obtained from energetics analysis are consistent with each other.

Overall, the semi-empirical approach-based calculations suggest that the preferred structure for monomers of KLVFFA and VQIVYKPVD is helical as they can be stabilized by many intramolecular hydrogen bonds. However, the monomer of QVEVKSEKLD exists preferentially as a linear structure due to favorable electrostatic contacts between the charged residues. The dimers of all the aforementioned peptide sequences show a double well potential, which indicates that they exist preferentially in β-sheet or α-helix forms where the former structures are stabilized by intermolecular hydrogen bonding while the latter structures are stabilized by intramolecular hydrogen bonds.

### 2.1. Energy Profile for Conformational Interconversion in Penta-Alanine System

**Figure 7:**
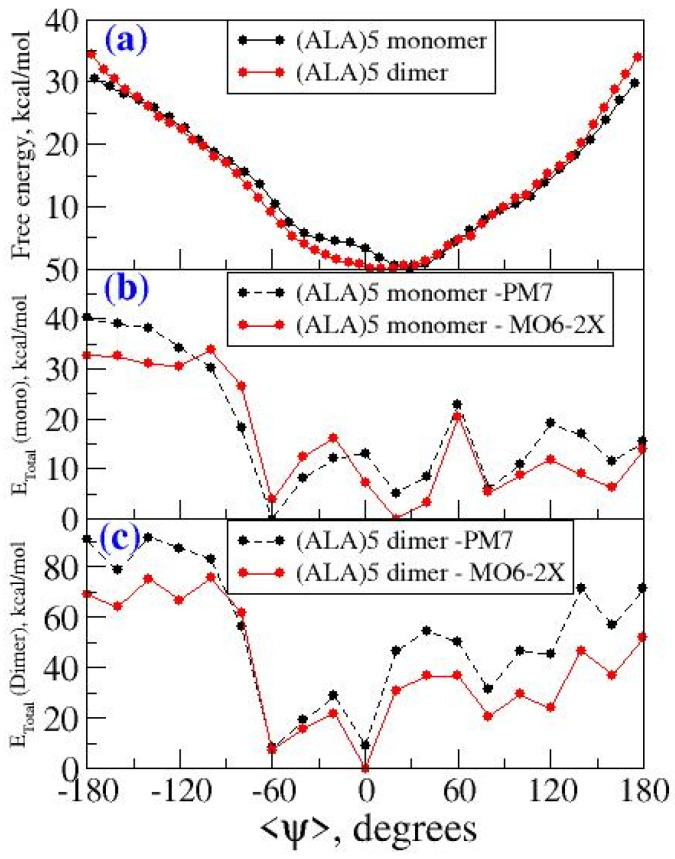
(**a**) Free energy profile as a function of <ψ> for penta-alanine in monomeric and dimeric form from wham analysis (**b**) Energy profile as a function of <ψ> for monomeric form of penta-alanine using PM7 and M06-2X level of theory (**c**) energy profile as a function of <ψ> for dimeric form of penta-alanine using PM7 and M06-2X level of theory.

**Figure 8:**
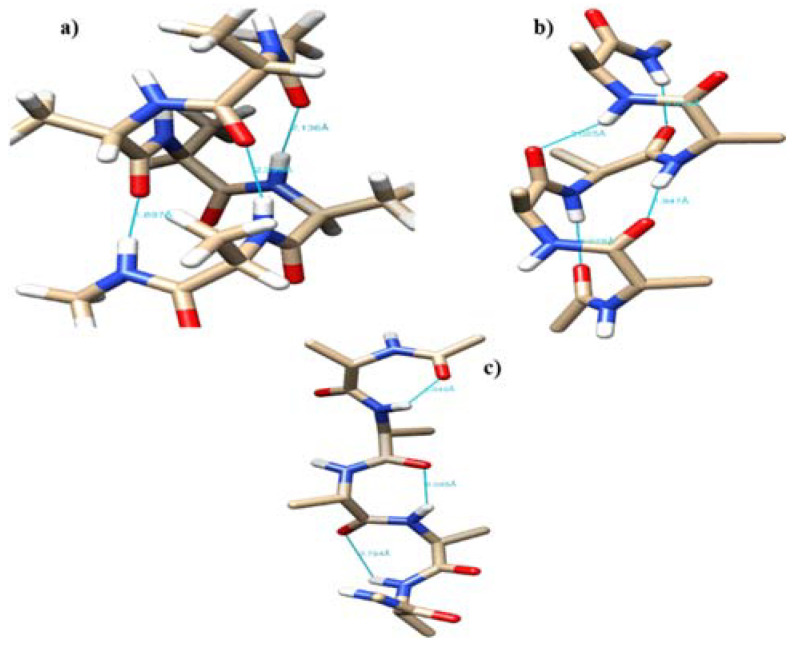
The representative configuration for penta-alanine monomer with (**a**) <ψ> = −60, (**b**) <ψ>=20, and (**c**) <ψ> = 80.

The free energy profile for conformational interconversion has been obtained using a WHAM analysis for both monomeric and dimeric forms of penta-alanine which has been considered as a control system. The results are shown in Figure 7. In contrast to the case of amyloid forming sequences, here the energy profile showed a shallow single minimum for the <ψ> angle in the range −30 °C and 30 °C. Further, the profile did not show any second minimum or shallow shoulder for the dihedral angle range 60–100 °C which generally correspond to beta-sheet like secondary structures. This indicates that penta-alanine remains in alpha-helix secondary structure in both monomeric and dimeric forms. The energy profiles computed using PM7 semi-empirical theory and M06-2X density functional theory also confirm the same results. The PM7 theory predicts a global minimum corresponding to <ψ> = −60 and the secondary structure corresponds to an alpha-helix with 3 intramolecular hydrogen bonds as shown in Figure 8a. Similarly, the M06-2X predicted a global minimum for the secondary structures having <ψ> = 20 which has 4 intramolecular hydrogen bonds (refer to Figure 8b). There was another local minimum observed for the <ψ> = 80 in the energy profile as predicted from the M06-2X level of theory and this corresponds to a linear structure (refer to Figure 8c) but interestingly this structure is also stabilized by 3 intramolecular hydrogen bonds. The structures shown in Figure 8a,b are higher in energy when compared to the structure shown in Figure 8c by about 4–5 kcal/mol.

### 2.2. Effect of Intrinsically Disordered Peptides (IDPs) Force Fields on Helix-to-Beta Interconversion Dynamics in Amyloid Forming Peptides

We emphasize that the reaction coordinate only allows the system to sample both alpha-helix and beta-sheet like secondary structures, but their relative energetics are dictated by the employed force-field and intrinsic nature of amyloid sequences. So, with the use of a force-field which rather stabilizes disordered structures, we should see change in the free energy profile and in particular the relative energetics of alpha-helix and beta-sheet like structures should be altered. To confirm this, we have carried out new sets of umbrella sampling simulations for monomer and dimer (antiparallel) of peptide sequences (KLVFFA) from amyloid beta protein using the ff99SB*-ILDN force-field [47,48] The calculated free energy profile is shown in Figure S8 and as it can be seen the monomer reveals a minimum at −30° ψ angle and at this minimum the peptide exists in coil and bend-like secondary structure (Table S4 and Figure S8a). However, in the antiparallel form, two minima appeared at <ψ> corresponding to −20° and 100° (Figure S4b,c). The secondary structure analysis revealed that at −20° the helix-like secondary structures dominate and at 100°, beta sheet-like structures are preferred. The free energy profile looked very similar to the case of dimeric peptide sequences from tau protein (QVEVKSEKLD and VQIVYKPVD). The ff99SB*-ILDN force-field derived free energy profile clearly reveals that the helix like structures are not very much stabilized in the monomer. In the case of antiparallel dimers, the alpha helix and beta-sheet like secondary structures are dominant, the same pattern has been observed with the use of FF03 force field. However, their relative energetics and so the relative population is altered considerably.

We have also analyzed the hydrogen bonding and secondary structures for the peptides along this reaction coordinate and included in the supporting information as Table S4 and Figure S8. These analyses when compared to previous case (i.e., the results obtained with the use of FF03) have shown that there is a reduced population of helix like structures for monomer peptides which reveals that the employed force-field certainly has an effect on the population of different secondary structures and their relative energetics.

## 3. Conclusions

Motivated by the fact that a wide variety of neurodegenerative diseases are characterized by the accumulation of protein aggregates in intraneuronal or extraneuronal brain regions, we have investigated the free energy profile of the amyloid forming peptide sequences from Aβ and tau proteins. Using umbrella sampling molecular dynamics with the <ψ> angle as a reaction coordinate, we have estimated the free energy changes associated with interconversion between different α-helix and β-sheet secondary structures. To further validate the free energy profiles obtained from the force-field approach, PM7 level semi-empirical calculations and density functional theory calculations at M06-2X/6-31+G* level of theory were carried out. The obtained energy profiles reveal that the KLVFFA and VQIVYKPVD sequences exist preferentially in helical form when they are in the monomeric state, while helical and β-sheet like secondary structures are predominant for the dimeric state. Analysis of hydrogen bonding and intermolecular energies indicated that the helical form is more stable in the monomer due to the possibility to form multiple intramolecular hydrogen bonds, while β-sheets are preferential in the dimeric form due to intermolecular hydrogen bonds. The semiempirical theory-based calculations yield similar results as the force-field methods, showing consistency between the energy profile for the monomer and intermolecular energy profiles for dimers with respect to average reaction coordinates. An interesting behavior for the QVEVKSEKLD monomer is that it preferentially exists in linear form. This can be attributed to the favorable electrostatic interactions between the charged residues that dominate over the energy gain from intramolecular hydrogen bonds feasible in α-helix like structures. As a control, we have also carried out umbrella sampling simulations for both monomeric and dimeric forms of penta-alanine. In this case, the free energy profile showed a single well corresponding to alpha-helix like secondary structures. It is known that poly-alanine prefers to exist in alpha-helix conformation. We have also studied the force-field effect on the free energy profile for the interconversion. The force-field developed for studying intrinsically disordered peptides (referred as IDP force-field) increases the presence of coil when compared to α-helix in monomer of KLVFFA. Further, in the case of dimer of this peptide in an antiparallel arrangement the IDP force field affects the free energy profile for interconversion and the population of α-helix and β-sheet conformers and their relative energetics. The current study shows that the interconversion dynamics depends not only on the amyloid sequences but also on the force-field used for the modeling. As we have shown in this study it is also further necessary to validate the free energy profile using more reliable electronic structure theory. The study on peptide sequences from tau protein reveals that energetics of secondary structures of monomers cannot be generalized that the α-helix structures are the most stable and has to be considered case by case while the energetics of dimers displayed a more general trend.

## 4. Materials and Methods

### 4.1. Computational Details

We carried out calculations for monomeric and dimeric (parallel and antiparallel) forms of amyloid forming sequences from amyloid-β and tau proteins. For the initial structure of monomeric and dimeric KLVFFA (16-21), we used the crystal structure with PDB id 5OQV based on recent cryogenic electron microscopy measurements where the structure for full-length amyloid-β fibril (1-42) has been reported [49] In this case, the dimer is arranged such that two monomers are parallel to each other. There is also a report of another crystal structure for the KLVFFA sequence (the reference PDB id is 3OW9) where the two monomers are arranged antiparallel to each other [50]. The peptides were capped with the Acetyl (CH_3_CO) group at the N-terminus and with the NMe (NHCH_3_) group at the C-terminus in order to avoid charge accumulation in the terminals. The individual calculations were performed on the monomer, and on the parallel and anti-parallel forms of the dimer. The monomer and dimer systems were studied in orthorhombic simulation boxes. The peptides were solvated with water molecules and neutralized by adding sufficient numbers of counter ions (Na^+^). In particular, the number of water molecules added was 3358, 2815 and 3460 respectively for monomer, dimer in parallel packing and dimer in antiparallel packing. The TIP3P [51] water model was used to describe the water solvent. The structures in solution were relaxed by energy minimization using the steepest descent method. Subsequently, simulations were carried out in a constant volume ensemble and finally, the simulations in the isothermal-isobaric ensemble were carried out at 300 K and 1 bar pressure. Temperature and pressure were controlled using the velocity rescaling and Parrinello-Rahman algorithms [52,53] The time step for the integration of the equation of motion was 1 fs and the time scale for the equilibration runs was 5 ns. All the classical molecular dynamics simulations were performed using the GROMACS-4.6 package [54,55,56] with the AMBER FF03 force field [57].

Similarly, the amyloid forming peptides from the tau protein with sequence QVEVKSEKLD (336-345) and VQIVYKPVD (306-314) were retrieved from the protein data bank (the PDB ID is 5O3O) [58] We extracted both monomeric and dimeric forms for these peptides and as described above a similar protocol was used to equilibrate these structures in water solvent. In this case, only dimers in the parallel configuration were investigated.

Further to understand the effect of force-fields on free energy profiles for helix to beta-sheet interconversion in monomer and dimeric (antiparallel) peptides, we have carried out new sets of calculations using the Amber ff99SB*-ILDN force field [47,48,59]. Umbrella sampling simulations using <ψ> as a reaction coordinate was carried out in the same way as we have described above.

### 4.2. Umbrella Sampling Simulations Using <ψ> as A Reaction Coordinate

The equilibrated structures from the classical molecular dynamics simulations were used as starting structures for subsequent umbrella sampling simulations. Similar to our previous studies [41] we have considered the <ψ> angle as a reaction coordinate. The reason for using <ψ> as a reaction coordinate is that it is practically simple but efficient to achieve the interconversion between α-helix and β-sheet like secondary structures. Another option is to use individual ψ (corresponding to each peptide bond) as a reaction coordinate and this means for a peptide like KLVFFA with 6 amino acids, the free energy profile will be six dimensional (f(ψ1, ψ2, ψ3, ψ4, ψ5)) and this is computationally very demanding to scan and also it will lead to complex multidimensional free energy surface leading to difficulties for carrying out analysis.

In the case of the monomer, the <ψ> angle of the KLVFFA is −170 °C, which is considered as the initial angle to start the calculations. In the case of the monomeric form of KLVFFA, there were 5 ψ angles in total with one corresponding to each peptide bond (Figure 1). We have carried out a total of 37 independent simulations with an incremental step of 10° and at each angle; we performed 10 ns simulations (a total of 370 nanoseconds). Overall, the simulations were carried out for the average ψ angle ranging from −180° to 180° with the 10° increment step. During these simulations, the angles were restrained using a harmonic spring constant of 150 kj mol^−1^ rad^−1^. All umbrella sampling simulations were performed using the PLUMED-2.0 package [60] During the simulations, the input configuration for each simulation was taken from the previous simulation to achieve equilibration quickly. The <ψ> angle distribution data and force-constants used for constraining the dihedral angles as obtained from the umbrella sampling simulation were used to calculate the free energy along with the <ψ> by employing a weighted histogram analysis method [61] (WHAM). A similar protocol was followed for parallel and antiparallel KLVFFA sequences where the <ψ> angles for the starting umbrella sampling simulations were kept at 130° and 150°, respectively. The reason for this is that we wanted to choose the starting <ψ> angle to be close to the values in the starting structure for the sequences. It is also notable that the number of dihedral angles constrained in the case of dimeric form is 10 as there are 10 possible peptide bonds. A similar procedure was followed for the two sequences from tau protein namely QVEVKSEKLD and VQIVYKPVD in both monomeric and dimeric forms. In the case of monomer, there were 9 dihedral angles restrained, while in the case of the dimeric form these dihedral angles are twice as many. The <ψ> angles of monomer and dimer forms of QVEVKSEKLD are 110° and VQIVYKPVD 120° respectively. As a control system penta-alanine was chosen and both monomeric and dimeric forms were studied. In the case of monomer, 5 dihedral angles (for dimer 10 dihedral angles) were constrained. The WHAM analysis was again employed to obtain the free energy profile along the reaction coordinate, <ψ>. Finally, the trajectories from the umbrella sampling simulations were further used for secondary structure and energy analysis and hydrogen bonding analysis. A selected number of independent configurations corresponding to simulations at a specific <ψ> angle were chosen for the QM calculations and the details are given below.

### 4.3. QM Calculations

We have extracted the coordinates for the peptides from the simulations with <ψ> angles in the range −180° to 180° with an increment of 20° In particular, for each angle, we used around 20 configurations. There are altogether 7 different systems (three corresponding to amyloid and 4 corresponding to tau) and peptide sequences were alone extracted from the trajectories after stripping out water solvents and ions. These geometries for the peptides were used to estimate the total energies at the PM7 semiempirical level of theory with the help of Gaussian 16 software [62], which are applicable for the large size of the peptide sequences. For the dimeric form of QVEVKSEKLD, we have also carried out DFT level calculations at M06-2X/6-31+G* level of theory [42] and number of configurations used for each dihedral angle were limited to 10 (instead of 20 as in the case of PM7). As we have shown, the energy profile as obtained from PM7 displayed similar behavior as the DFT level of theory. Moreover, from a previous study, we learned that the semiempirical level of theory can provide comparable results for the energetics of amyloid forming sequences and their mutant variants when compared to B3LYP density functional theory [41] based calculations. The calculations were single point energy calculations and geometry optimizations were not performed. We followed this procedure since optimization will take the peptides to the nearest minimum in the potential energy landscape while our intention is to get the full potential energy profile along the reaction coordinate (i.e., for all values of <ψ> from −180° to 180°) including the barriers if there are any. Finally, the total energies for all 20 configurations corresponding to each <ψ> value were averaged and subtracted with the energy value corresponding to the minimum so that the base value for all the plots was set to zero. The QM calculations at PM7 level and M06-2X level were also carried out for the monomeric and dimeric forms of penta-alanine. In this case, 10 configurations for each dihedral angle chosen at the intervals of 20° were used for the calculations.

## Data Availability

Data sharing not applicable.

## Supplementary Materials

The following are available online at https://www.mdpi.com/1422-0067/22/6/3244/s1. Table S1. Secondary structure elements of monomeric and dimeric forms (in both parallel and antiparallel packing) of KLVFFA sequence. Table S2. Secondary structure elements of monomeric and dimeric forms (in parallel packing) of QVEVKSEKLD. Table S3. Secondary structure elements of monomeric and dimeric forms of VQIVYKPVD sequence. Table S4. Secondary structure elements of monomeric and dimeric forms (in antiparallel packing) of KLVFFA using Amber ff99sb*-ildn force field. Figure S1. Representative conformers for the KLVFFA monomer corresponding to the flat minimum in free energy profile. The yellow dots represent the intramolecular hydrogen bonding. Figure S2. RMSD plots of Cα atoms of KLVFFA monomer and dimeric forms from umbrellas sampling simulations. (a) Monomer (b) Dimeric form in parallel packing (c) Dimeric form in antiparallel packing. Figure S3. Probability distributions of dihedral angles for KLVFFA monomer along the free energy profile. Figure S4. Average number of hydrogen bonds in monomer, and average number of intra and intermolecular hydrogen bonds in dimeric forms (in parallel and antiparallel packing) of KLVFFA respectively. Figure S5. Average number of hydrogen bonds in monomer, and average number of intra and intermolecular hydrogen bonds in dimer of QVEVKSEKLD and VQIVYKPVD respectively. Figure S6. Total and inter-molecular energies of (a) Monomer of KLVFFA and (b) Parallel and antiparallel forms of KLVFFA. Figure S7. Total and inter molecular energies of (a) Monomer of QVEVKSEKLD and VQIVYKPVD (b) Dimeric forms of QVEVKSEKLD and VQIVYKPVD. Figure S8. Free energy profile for monomer and dimeric forms of KLVFFA using Amber ff99sb*-ildn force field and representative conformations from umbrella sampling MD. (a) coil form of monomer, (b) and (c) helical and β-sheet forms of dimer (anti-parallel form). Figure S9. Average number of hydrogen bonds in monomer, and average number of intra and intermolecular hydrogen bonds in dimeric form (antiparallel packing) of KLVFFA respectively obtained using Amber ff99sb*-ildn force field.

## Abbreviations

AD	Alzheimer’s disease
PD	Parkinson’s disease
HD	Huntington’s diseases
ALS	Amyotrophic Lateral Sclerosis
FTLD	Frontotemporal lobar degeneration
WHAM	Weighted Histogram Analysis Method
MD	Molecular Dynamics
REMD	Replica-Exchange Molecular Dynamics
DSSP	Dictionary of Secondary Structure of Proteins
DFT	Density Functional Theory
QM	Quantum mechanics
RMSD	Root Mean Square Deviation
