# Supplementary material for: Assessment of Amyloid Forming Tendency of Peptide Sequences from Amyloid Beta and Tau Proteins Using Force-Field, Semi-Empirical, and Density Functional Theory Calculations
Source: Int J Mol Sci. 2021 Mar 23;22(6):3244. doi: 10.3390/ijms22063244 (PMC8005029; doi:10.3390/ijms22063244)
Supplement: Supplementary file 1 [file ijms-22-03244-s001.pdf]

Assessment of Amyloid Forming Tendency of Peptide Sequences from Amyloid beta and Tau proteins using Force-field, Semi-empirical and Density Functional Theory Calculations

Charuvaka Muvva [1,2,3] Natarajan Arul Murugan [1,*] and Venkatesan Subramanian [2,3,4]

1 Division of Theoretical Chemistry and Biology, School of Engineering Sciences in Chemistry, Biotechnology and Health, KTH Royal Institute of Technology, S-106 91 Stockholm, Sweden.

2 Inorganic & Physical Chemistry Laboratory, CSIR-Central Leather Research Institute, Adyar, Chennai 600020, India.

3 Academy of Scientific and Innovative Research (AcSIR), Ghaziabad- 201002, India.

4 Centre for High Computing, CSIR-CLRI, Adyar, Chennai, Tamilnadu, 600 020, India.

*E-mail: murugan@kth.se

Table S1. Secondary structure elements of monomeric and dimeric forms (in both parallel and antiparallel packing) of KLVFFA sequence.

Monomer					Parallel				Antiparallel			
**Angles**	**Coil**	**Bend**	**A-Helix**	**B-Sheet**	**Coil**	**Bend**	**A-Helix**	**B-Sheet**	**Coil**	**Bend**	**A-Helix**	**B-Sheet**
**-180**	1	-	-	-	0.86	-	-	0.14	1	-	-	-
**-170**	1	-	-	-	0.96	-	-	0.04	0.97	0.01	-	0.02
**-160**	1	-	-	-	1	-	-	-	0.98	0.01	-	0.01
**-150**	0.98	0.02	-	-	1	-	-	-	1	-	-	-
**-140**	0.98	0.02	-	-	0.93	0.07	-	-	0.98	0.01	-	-
**-130**	0.99	0.01	-	-	0.83	0.15	-	0.02	0.96	0.03	-	-
**-120**	0.95	0.05	-	-	0.81	0.17	-	0.02	0.87	0.12	-	-
**-110**	0.83	0.17	-	-	0.75	0.25	-	-	0.73	0.26	-	-
**-100**	0.74	0.26	-	-	0.67	0.31	0.02	-	0.70	0.29	-	-
**-90**	0.67	0.33	-	-	0.49	0.16	0.35	-	0.59	0.24	0.17	-
**-80**	0.33	-	0.67	-	0.33	-	0.67	-	0.35	-	0.65	-
**-70**	0.66	0.33	0.01	-	0.33	-	0.67	-	0.33	-	0.67	-
**-60**	0.66	0.32	0.02	-	0.33	-	0.67	-	0.38	-	0.62	-
**-50**	0.67	0.33	-	-	0.34	-	0.66	-	0.34	-	0.66	-
**-40**	0.34	-	0.66	-	0.34	-	0.66	-	0.35	-	0.65	-
**-30**	0.37	-	0.63	-	0.37	-	0.63	-	0.35	-	0.65	-
**-20**	0.36	-	0.64	-	0.41	0	0.59	-	0.33	-	0.67	-
**-10**	0.37	-	0.63	-	0.40	-	0.60	-	0.40	0	0.60	-
**0**	0.49	0.01	0.50	-	0.44	-	0.56	-	0.44	0	0.56	-
**10**	0.62	0.06	0.32	-	0.51	-	0.49	-	0.55	0.02	0.43	-
**20**	0.76	-	0.24	-	0.71	0.01	0.28	-	0.66	0	0.34	-
**30**	0.85	0.01	0.14	-	0.80	0	0.20	-	0.79	-	0.21	-
**40**	0.83	0.01	0.16	-	0.94	0.01	0.01	0.04	0.86	0	0.14	-
**50**	0.9	-	0.1	-	0.95	-	0.02	0.03	0.95	0	0.03	0.02
**60**	0.98	-	0.02	-	0.95	-	-	0.05	0.98	-	-	0.02
**70**	1	-	-	-	0.73	-	-	0.27	0.78	-	-	0.22
**80**	0.99	0.01	-	-	0.48	-	-	0.52	0.44	-	-	0.56
**90**	1	-	-	-	0.37	-	-	0.63	0.41	-	-	0.59
**100**	1	-	-	-	0.34	-	-	0.66	0.43	-	-	0.57
**110**	1	-	-	-	0.35	-	-	0.65	0.36	-	-	0.64
**120**	1	-	-	-	0.35	-	-	0.65	0.38	-	-	0.62
**130**	1	-	-	-	0.39	-	-	0.61	0.43	-	-	0.57
**140**	1	-	-	-	0.42	-	-	0.58	0.41	-	-	0.59
**150**	1	-	-	-	0.5	-	-	0.50	0.35	-	-	0.65
**160**	1	-	-	-	0.61	-	-	0.39	0.52	-	-	0.48
**170**	1	-	-	-	1	-	-	-	0.33	-	-	0.67
**180**	1	-	-	-	0.79	0.01	-	0.20	0.48	-	-	0.52

Table S2. Secondary structure elements of monomeric and dimeric forms (in parallel packing) of QVEVKSEKLD.

Monomer					parallel			
**Angles**	**Coil**	**Bend**	**A-Helix**	**B-Sheet**	**Coil**	**Bend**	**A-Helix**	**B-Sheet**
**-180**	1	-	-	-	0.43	0.07	-	0.49
**-170**	1	-	-	-	0.41	0.03	0.01	0.55
**-160**	1	-	-	-	0.41	0.03	0.01	0.55
**-150**	1	-	-	-	0.64	0.04	0	0.32
**-140**	1	-	-	-	0.90	0.06	-	0.04
**-130**	1	-	-	-	0.93	0.05	-	0.02
**-120**	1	-	-	-	0.93	0.07	-	-
**-110**	0.93	0.01	0.06	-	0.90	0.08	0.02	0
**-100**	0.71	0.04	0.25	-	0.69	0.16	0.15	-
**-90**	0.52	0.08	0.4	-	0.50	0.11	0.39	-
**-80**	0.41	0.09	0.5	-	0.42	0.07	0.51	-
**-70**	0.31	0.01	0.68	-	0.31	0.07	0.62	-
**-60**	0.29	0.04	0.67	-	0.33	0.08	0.59	-
**-50**	0.28	0.02	0.70	-	0.29	0.03	0.68	-
**-40**	0.23	-	0.77	-	0.30	0.01	0.69	-
**-30**	0.2	-	0.80	-	0.29	0.01	0.70	-
**-20**	0.2	-	0.8	-	0.26	0.02	0.71	-
**-10**	0.2	-	0.80	-	0.24	0	0.76	-
**0**	0.2	-	0.8	-	0.21	0.02	0.77	-
**10**	0.2	0	0.8	-	0.23	0.02	0.75	-
**20**	0.32	0.15	0.53	-	0.42	0.20	0.38	-
**30**	0.43	0.54	0.03	-	0.51	0.45	0.04	-
**40**	0.5	0.5	-	-	0.55	0.45	-	-
**50**	0.62	0.38	-	-	0.6	0.4	-	-
**60**	0.6	0.4	-	-	0.6	0.4	-	-
**70**	0.62	0.38	-	-	0.54	0.46	-	-
**80**	0.64	0.36	-	-	0.56	0.44	-	-
**90**	0.66	0.34	-	-	0.56	0.44	-	-
**100**	0.61	0.39	-	-	0.58	0.4	-	0.02
**110**	0.72	0.28	-	-	0.65	0.32	-	0.03
**120**	1	-	-	-	0.61	-	-	0.39
**130**	1	-	-	-	0.68	-	-	0.32
**140**	1	-	-	-	0.69	-	-	0.31
**150**	1	-	-	-	0.68	-	-	0.32
**160**	1	-	-	-	0.83	-	-	0.17
**170**	1	-	-	-	0.81	-	-	0.19
**180**	1	-	-	-	0.81	-	-	0.19

Table S3. Secondary structure elements of monomeric and dimeric forms of VQIVYKPVD sequence.

Monomer					parallel			
**Angles**	**Coil**	**Bend**	**A-Helix**	**B-Sheet**	**Coil**	**Bend**	**A-Helix**	**B-Sheet**
**-180**	0.99	0.01	-	-	0.57	-	-	0.43
**-170**	1	-	-	-	0.55	-	-	0.45
**-160**	1	-	-	-	0.63	-	-	0.37
**-150**	1	-	-	-	0.58	-	-	0.42
**-140**	1	-	-	-	0.57	0.03	0.03	0.37
**-130**	1	-	-	-	0.86	0.04	0.03	0.07
**-120**	1	-	-	-	0.93	0.04	0.01	0.02
**-110**	0.96	0.01	0.03	-	0.94	0.05	-	-
**-100**	0.91	0.02	0.07	-	0.92	0.05	0.03	-
**-90**	0.75	0.01	0.24	-	0.78	0.06	0.16	-
**-80**	0.52	0.08	0.40	-	0.65	0.08	0.27	-
**-70**	0.46	0.08	0.46	-	0.50	0.06	0.44	-
**-60**	0.4	0.1	0.50	-	0.42	0.06	0.52	-
**-50**	0.31	0.04	0.65	-	0.35	0.07	0.58	-
**-40**	0.3	0.04	0.66	-	0.32	0.05	0.63	-
**-30**	0.27	0.04	0.69	-	0.31	0.06	0.63	-
**-20**	0.24	0.02	0.74	-	0.30	0.02	0.68	-
**-10**	0.22	-	0.78	-	0.28	0	0.72	-
**0**	0.22	-	0.78	-	0.27	-	0.73	-
**10**	0.23	-	0.77	-	0.29	0.02	0.69	-
**20**	0.29	0.03	0.68	-	0.34	0.07	0.59	-
**30**	0.3	0.16	0.54	-	0.38	0.13	0.49	-
**40**	0.46	0.44	0.10	-	0.46	0.4	0.14	-
**50**	0.46	0.52	0.02	-	0.48	0.48	0.04	-
**60**	0.47	0.53	-	-	0.48	0.5	0.02	-
**70**	0.5	0.5	-	-	0.58	0.41	-	-
**80**	0.57	0.43	-	-	0.61	0.38	-	-
**90**	0.6	0.4	-	-	0.62	0.37	-	-
**100**	0.62	0.38	-	-	0.65	0.34	-	-
**110**	0.69	0.31	-	-	0.64	0.35	-	-
**120**	0.77	0.23	-	-	0.69	0.30	-	-
**130**	1	-	-	-	0.56	-	-	0.43
**140**	1	-	-	-	0.55	-	-	0.44
**150**	1	-	-	-	0.63	0	-	0.36
**160**	1	-	-	-	0.62	-	-	0.38
**170**	0.99	0.01	-	-	0.88	-	-	0.12
**180**	0.98	0.02	-	-	1	-	-	-

Table S4. Secondary structure elements of monomeric and dimeric forms (in antiparallel packing) of KLVFFA using Amber ff99sb*-ildn force field.

Monomer					Antiparallel			
**Angles**	**Coil**	**Bend**	**A-Helix**	**B-Sheet**	**Coil**	**Bend**	**A-Helix**	**B-Sheet**
**-180**	1	-	-	-	0.87	0.13	-	-
**-170**	1	-	-	-	0.88	0.12	-	-
**-160**	1	-	-	-	0.84	0.16	-	-
**-150**	1	-	-	-	0.83	0.17	-	-
**-140**	0.99	0.01	-	-	0.79	0.21	-	-
**-130**	0.98	0.02	-	-	0.72	0.28	-	-
**-120**	0.94	0.06	-	-	0.72	0.28	-	-
**-110**	0.80	0.20	-	-	0.72	0.28	-	-
**-100**	0.68	0.32	-	-	0.72	0.28	-	-
**-90**	0.67	0.33			0.67	0.33	-	-
**-80**	0.61	0.39			0.53	0.43	0.04	-
**-70**	0.55	0.45			0.39	0.12	0.49	-
**-60**	0.50	0.50			0.39	0.04	0.57	-
**-50**	0.48	0.52			0.36	0.08	0.56	-
**-40**	0.47	0.53			0.38	0.19	0.43	-
**-30**	0.43	0.57	-	-	0.37	0.19	0.44	-
**-20**	0.40	0.60	-	-	0.36	0.16	0.48	-
**-10**	0.61	0.25	-	-	0.40	0.24	0.36	-
**0**	0.77	0.23	-	-	0.43	0.32	0.25	-
**10**	0.74	0.26	-	-	0.55	0.28	0.17	-
**20**	0.67	0.33	-	-	0.66	0.24	0.10	-
**30**	0.75	0.25	-	-	0.78	0.22	-	-
**40**	0.67	0.33	-	-	0.94	-	0.06	-
**50**	0.64	0.36	-	-	1	-	-	-
**60**	0.81	0.19	-	-	1	-	-	-
**70**	0.74	0.26	-	-	1	-	-	-
**80**	0.79	0.21	-	-	0.77	-	-	0.23
**90**	0.80	0.20	-	-	0.65	-	-	0.35
**100**	0.98	0.02	-	-	0.83	-	-	0.17
**110**	1	-	-	-	0.74	-	-	0.26
**120**	1	-	-	-	0.74	-	-	0.26
**130**	1	-	-	-	0.78	-	-	0.22
**140**	1	-	-	-	0.79	-	-	0.21
**150**	1	-	-	-	0.73	-	-	0.27
**160**	1	-	-	-	0.55	-	-	0.45
**170**	1	-	-	-	0.62	-	-	0.38
**180**	1	-	-	-	0.64	-	-	0.36

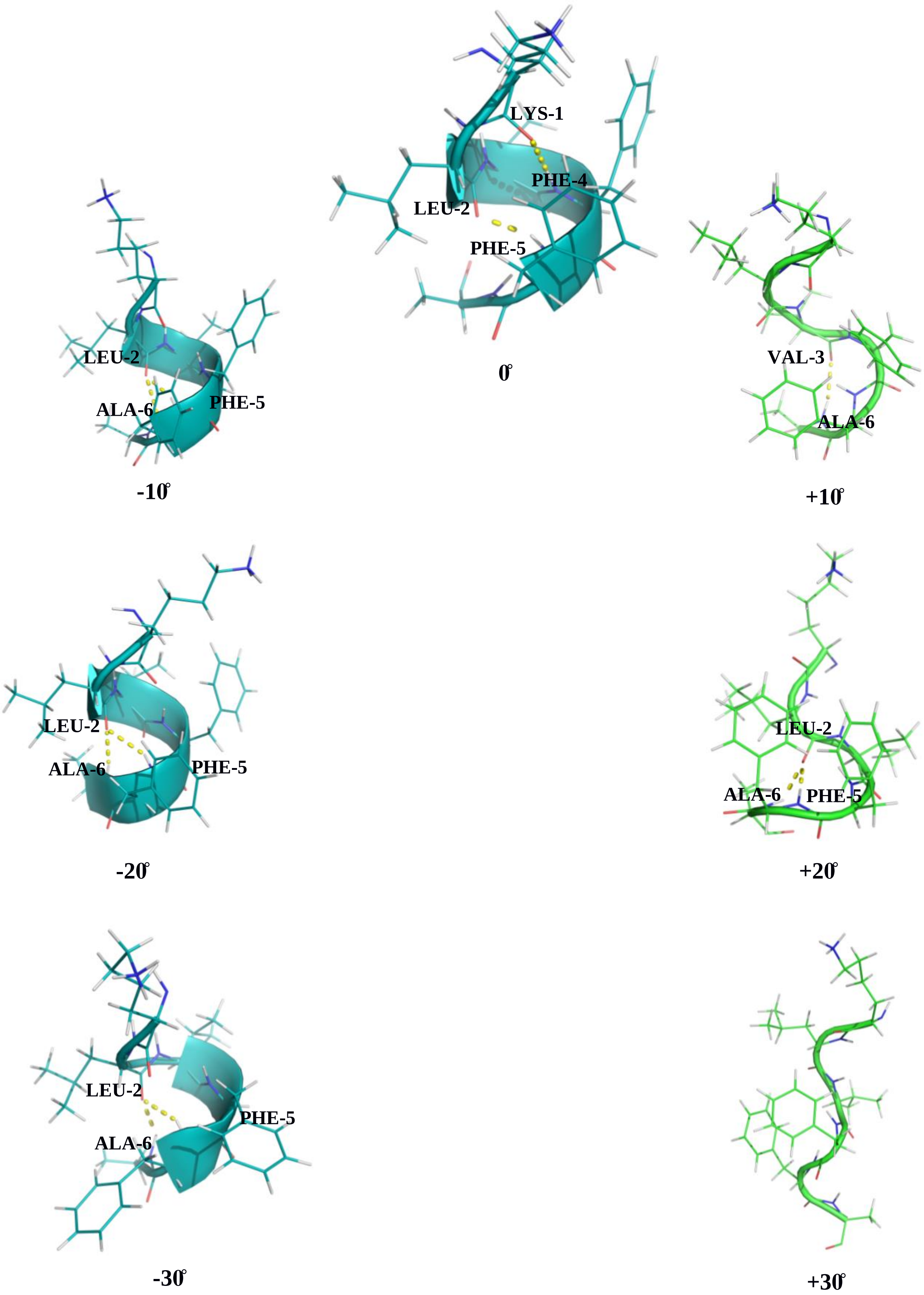

Figure S1. Representative conformers for the KLVFFA monomer corresponding to the flat minimum in free energy profile. The yellow dots represent the intramolecular hydrogen bonding.

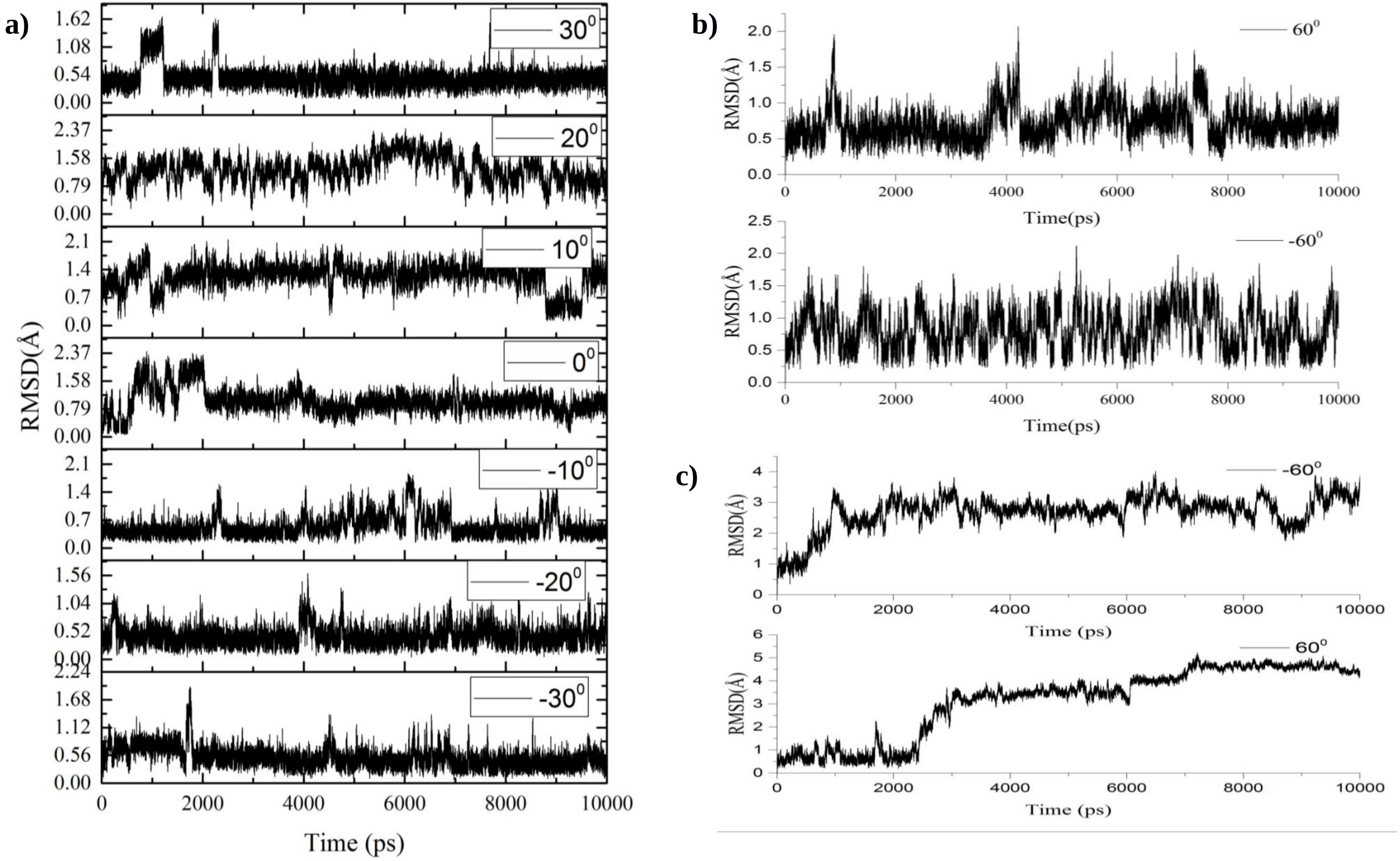

Figure S2. RMSD plots of Cα atoms of KLVFFA monomer and dimeric forms from umbrellas sampling simulations. (a) Monomer (b) Dimeric form in parallel packing (c) Dimeric form in antiparallel packing.

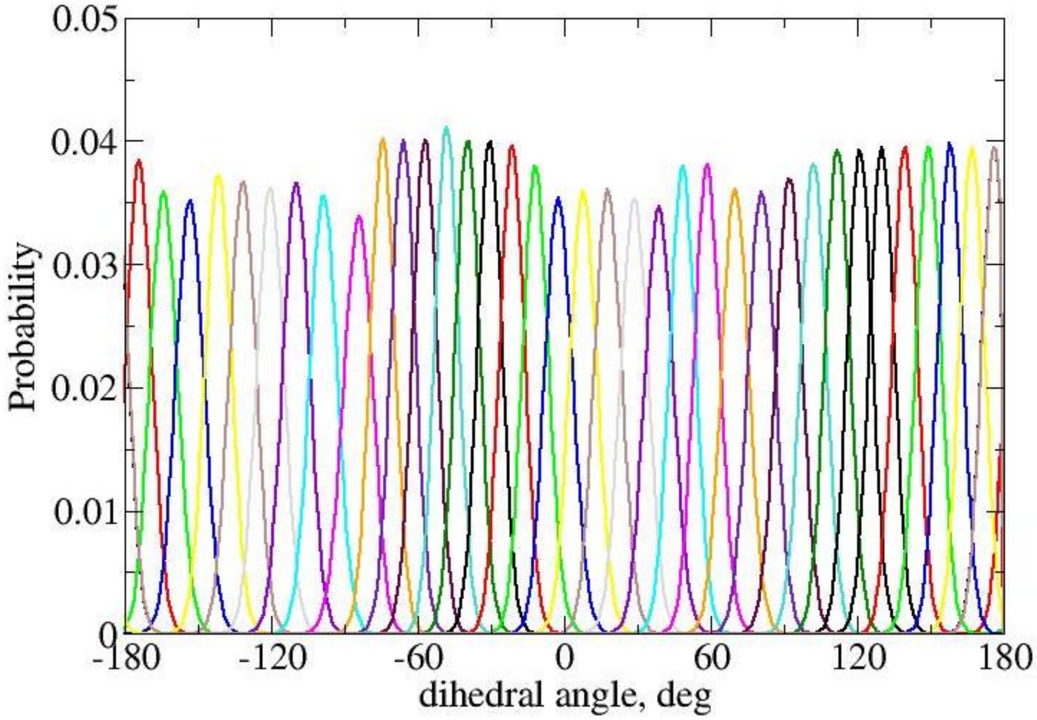

Figure S3. Probability distributions of dihedral angles for KLVFFA monomer along the free energy profile.

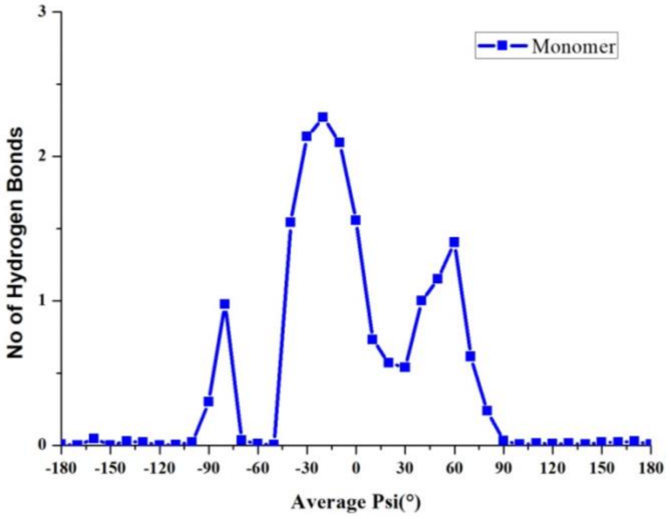

Monomer

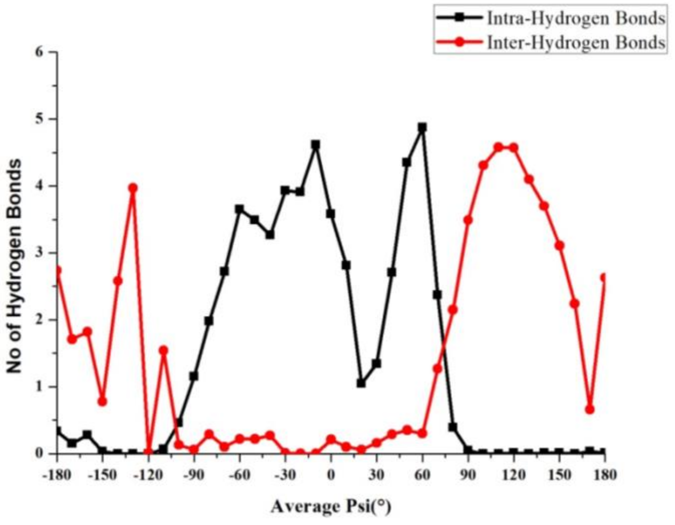

Parallel

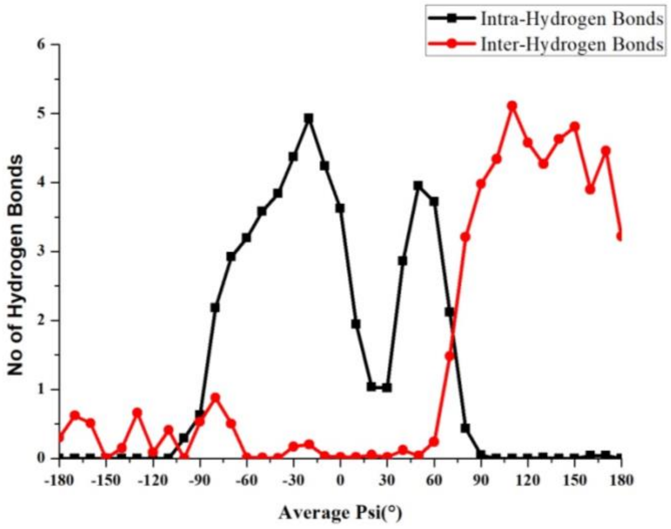

AntiParallel

Figure S4. Average number of hydrogen bonds in monomer, and average number of intra and intermolecular hydrogen bonds in dimeric forms (in parallel and antiparallel packing) of KLVFFA respectively.

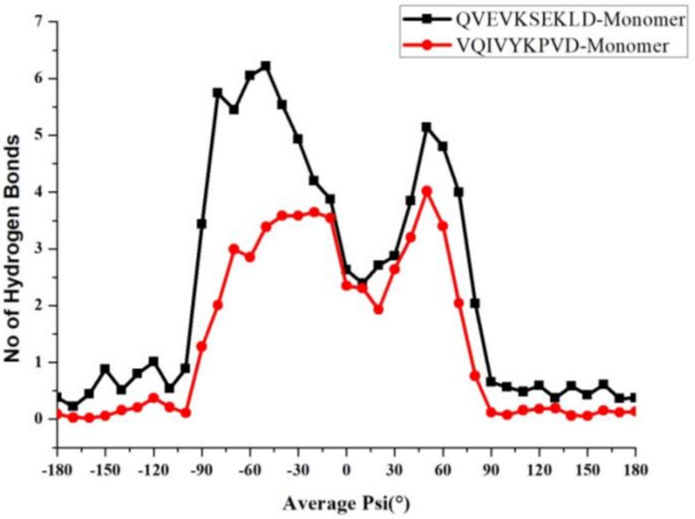

Monomer of QVEVKSEKLD and VQIVYKPVD

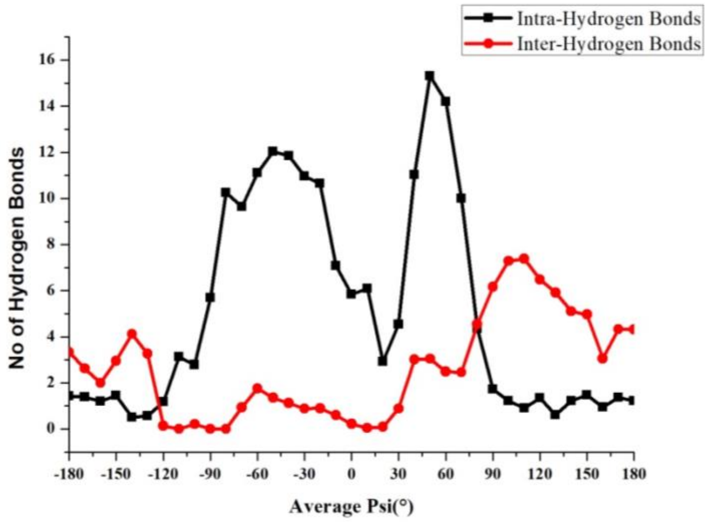

QVEVKSEKLD-Parallel

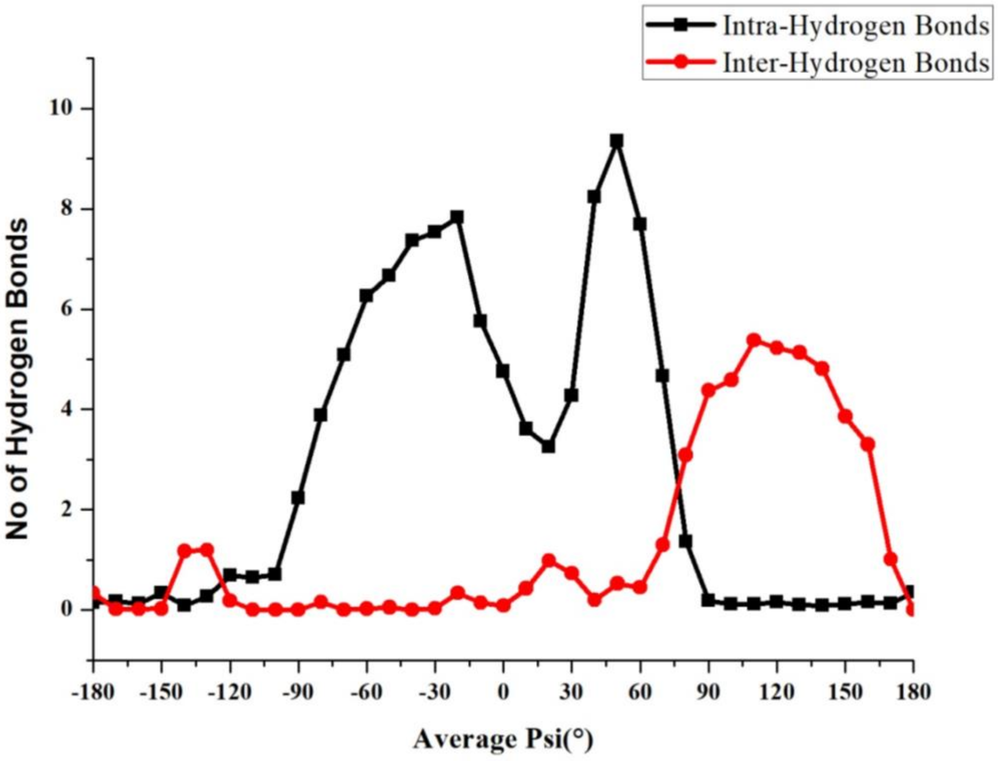

VQIVYKPVD-Parallel

Figure S5. Average number of hydrogen bonds in monomer, and average number of intra and intermolecular hydrogen bonds in dimer of QVEVKSEKLD and VQIVYKPVD respectively.

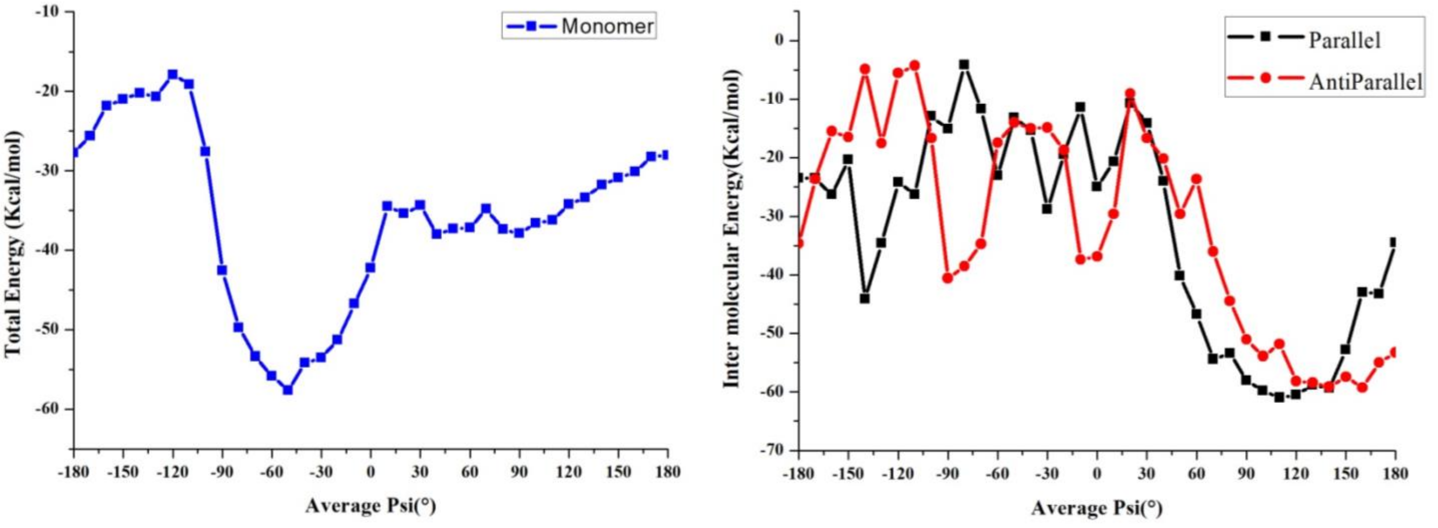

a). Monomer of KLVFFA

b). Parallel & Antiparallel of KLVFFA

Figure S6. Total and inter-molecular energies of (a) Monomer of KLVFFA and (b) Parallel and antiparallel forms of KLVFFA.

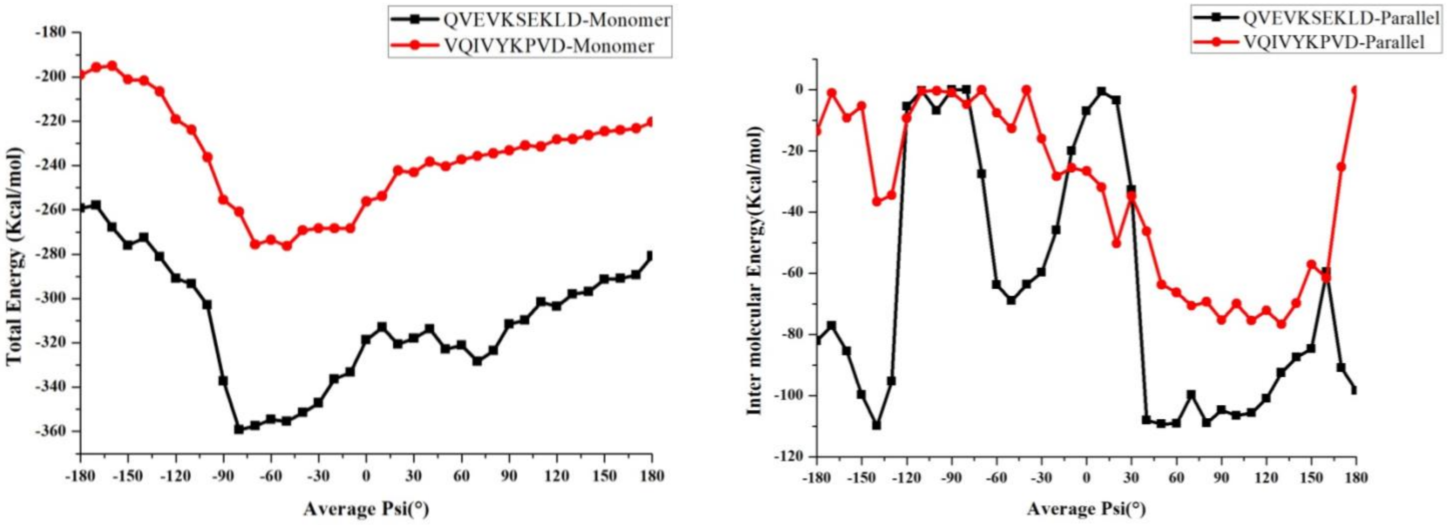

a). Monomer forms of QVEVKSEKLD and VQIVYKPVD

b). Dimer forms of QVEVKSEKLD and VQIVYKPVD

Figure S7. Total and inter molecular energies of (a) Monomer of QVEVKSEKLD and VQIVYKPVD (b) Dimeric forms of QVEVKSEKLD and VQIVYKPVD.

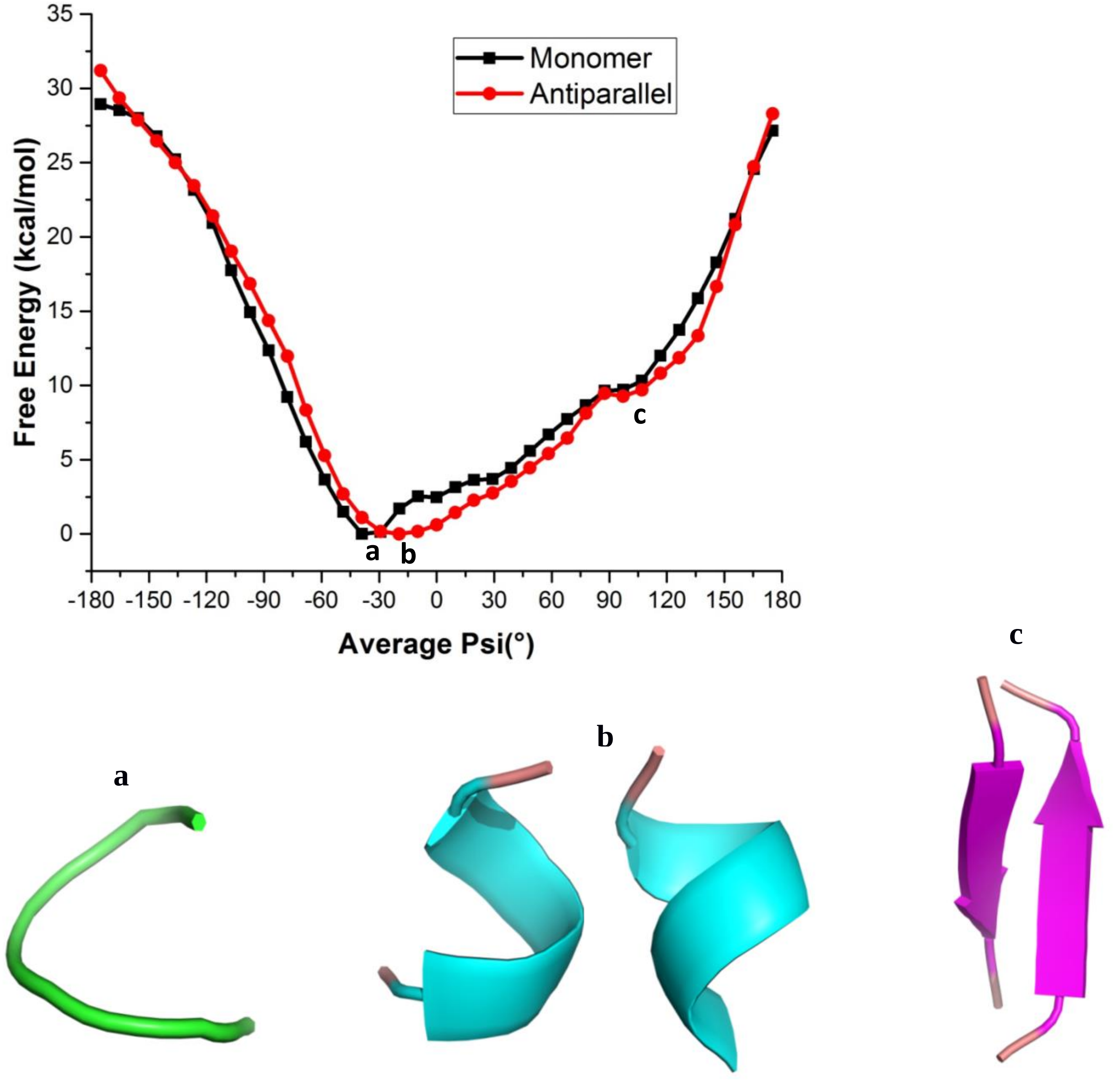

Figure S8. Free energy profile for monomer and dimeric forms of KLVFFA using Amber ff99sb*-ildn force field and representative conformations from umbrella sampling MD. (a) coil form of monomer, (b) and (c) helical and β-sheet forms of dimer (anti-parallel form)

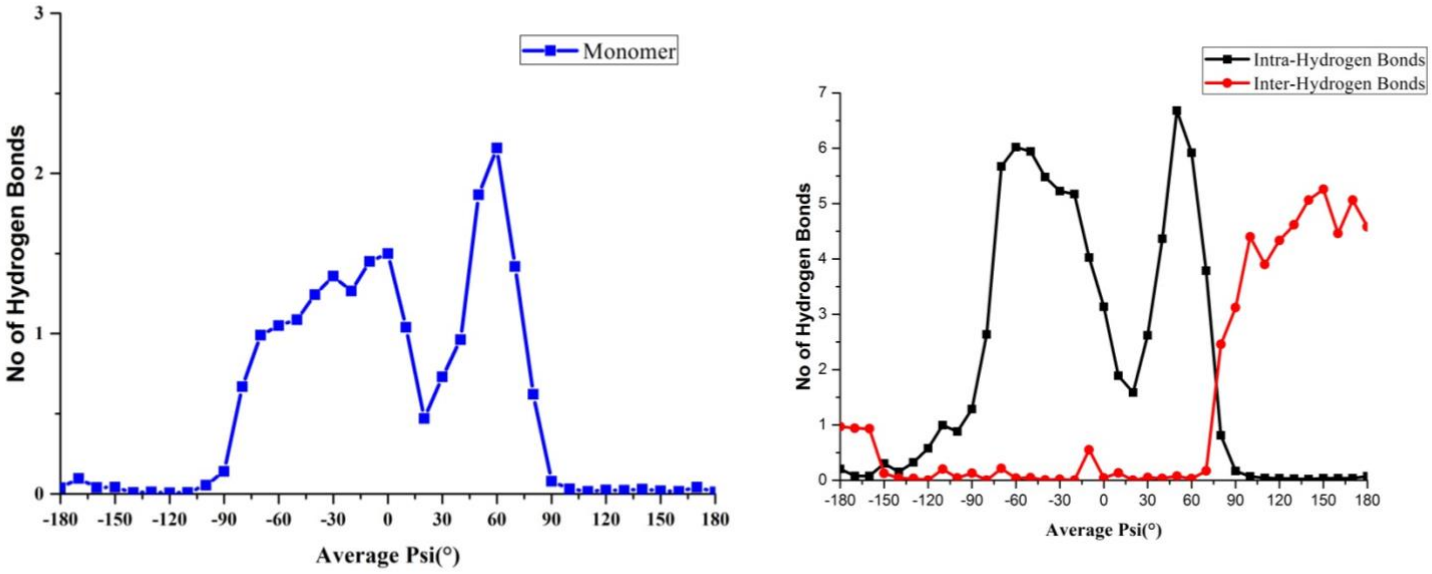

Figure S9. Average number of hydrogen bonds in monomer, and average number of intra and intermolecular hydrogen bonds in dimeric form (antiparallel packing) of KLVFFA respectively obtained using Amber ff99sb*-ildn force field.
